# Research on Factors Affecting Asphalt Mixtures’ Resistance to High-Frequency Freeze-Thaw in Plateau Areas

**DOI:** 10.3390/ma18030640

**Published:** 2025-01-31

**Authors:** Jinmei Wang, Jin Yang, Wenqi Wang, Bai Li, Chengjun He, Long He, Yalin Li

**Affiliations:** School of Architecture and Civil Engineering, Xihua University, Chengdu 610039, China; 1220210013@xhu.edu.cn (J.W.); libai@stu.xhu.edu.cn (B.L.); hechengjun@stu.xhu.edu.cn (C.H.); helong@stu.xhu.edu.cn (L.H.); liyalin@stu.xhu.edu.cn (Y.L.)

**Keywords:** asphalt pavement materials, high-frequency freeze-thaw, modified asphalt, asphalt mixtures, plateau area

## Abstract

Aiming at the problem that asphalt pavement materials in plateau areas are vulnerable to freeze-thaw damage, research was carried out on asphalt pavements of representative road sections, and the temperature within the pavement structure was monitored using buried sensors. Based on this, an indoor test method for high-frequency freeze-thaw was established, and UV, thermo-oxygen-aging and high-frequency freeze-thaw tests were combined. The effects of aging and maximum aggregate particle size on the resistance of asphalt mixtures to high-frequency freeze-thaw were investigated using the splitting strength ratio, mass-loss rate and void-ratio changes by employing the newly made RS-type modified asphalt in the laboratory. At the same time, the high-frequency freeze-thaw resistance of the asphalt mixture was compared with that of the SS/SMA-13 asphalt mixture on the top layer of a representative road section. The results show that UV aging at 180 h followed by thermal-oxygen aging at 120 h has the greatest impact on the asphalt mixture; in this condition, the high-frequency freeze-thaw-cycle asphalt mixture with freeze-thaw damage is affected by the rule of change of the third-degree polynomial. In the plateau environment conditions, compared with the original pavement material (SS-type modified asphalt), the RS-type modified asphalt has better anti-aging properties, adhesion properties and elasticity performance.

## 1. Introduction

In highland areas (average altitude above 3000 m), solar ultraviolet radiation is stronger due to the thinner atmosphere, which contrasts with the situation at lower altitudes [1,2,3]. Asphalt mixtures used in plateau areas tend to become hard and brittle after prolonged UV exposure, which greatly increases the risk of low temperatures and fatigue cracking [4,5]. Therefore, UV oxidation poses a major threat to the durability of asphalt pavement materials in these regions and has attracted urgent and critical attention from relevant researchers [6]. Another noteworthy environmental factor is the frequent freeze-thaw (F-T) cycles caused by temperature and humidity fluctuations, which exacerbate the damage to asphalt pavement materials [7]. With F-T damage and traffic loads, cracks and defects gradually appear in the internal structure of asphalt mixtures [8]. These cracks seriously damage the integrity of the asphalt surface layer and the waterproof bonding layer, allowing water to penetrate into the pavement base, thus affecting the durability of the road structure in mountainous regions of the plateau [9,10].

Existing research results on the F-T resistance of asphalt pavement materials in foreign countries focus on the effects of water, air, temperature, number of F-T cycles and other factors on the macroscopic performance indexes of road engineering materials. Huang and other researchers conducted a study on the effects of F-T cycles on AC- and SMA-graded asphalt mixtures, and the results showed that SMA asphalt mixtures had better F-T resistance performance [11]. You used the interfacial bond strength test to evaluate the effect of F-T cycling on the durability of asphalt mixtures, taking into account the changes in the bond between aggregate and asphalt during the F-T cycle [12]. Lachance focused on the viscoelastic properties of modified asphalt mixtures under F-T cycling and investigated the effect of aggregates on the viscoelastic properties based on complex modulus tests [13]. Hong et al. tested the dynamic modulus and mass loss of cold pavements under F-T cycles and evaluated the strain–stress behavior of the specimens to analyze their F-T durability [14]. Kavussi used rubber particles to modify asphalt mixtures and evaluated their water stability and fatigue performance under F-T cycling based on the response surface method, and the results showed that rubber particles were more effective at improving the fatigue performance of asphalt mixtures under F-T cycling [15]. Chun H, in order to better understand the mechanical behavior of asphalt mixtures under the action of F-T cycle loading, employed the generalized power law function, Prony series function and Burgers model for comparative analysis, and the results show that the Prony series function can be better predicted under the action of the F-T of asphalt mixtures under the relaxation modulus [16]. Das B studied the effect of two-stage aging and three-stage moisture conditioning on the fatigue damage of asphalt mixtures, and by measuring the modulus of strength and fatigue life of asphalt mixtures at 15 °C and 25 °C, it was found that the modulus of strength was increased with the increase in the degree of aging, and the fatigue life of asphalt mixtures was reduced by F-T [17]. Lvqvist L established an F-T damage model for asphalt mixtures based on thermodynamic theory, which considered the coupling effect of F-T damage and elastic-strain energy damage at the same time, and they proved that freezing time and low temperature are the main factors that cause F-T damage [18]. Anxin M explored the feasibility of F-T damage characteristics of asphalt mixtures by using two nondestructive testing techniques, namely the free-resonance testing method and the Rayleigh wave. The results showed that the second-order damping ratio was linearly related to the degree of F-T damage and could evaluate the F-T damage status of asphalt mixtures [19]. Zeyu Z studied the damage law of water-saturated asphalt mixtures under F-T conditions from the point of view of damage mechanics, and the results of the indoor F-T cycle test showed that the change of the void ratio of the mixture specimen can characterize the nonlinear process of damage within the specimen [20].

Related researchers have also conducted considerable research analyzing the performance of asphalt after aging. Gang Liu and Erilc Nielsen investigated the aging pattern of polymer-modified asphalt at different times as well as on different occasions by comparing the indoor aging test with the outdoor field-aging test. The results showed that the asphalt viscosity was higher after 22 years of field aging compared to the indoor viscosity of the asphalt after 9 days of aging, and the asphaltene content of the asphaltene was higher in indoor than in outdoor aging [21]. Young. S. Doh and Soon-Jea Lee analyzed the molecular weight distribution of modified asphalt before and after aging by gel chromatography, and the GPC spectra showed that matrix asphalt peaks shifted to the left while the SBS peaks shifted to the right, i.e., the molecular weight of the matrix asphalt increased while that of the SBS decreased. The authors concluded that the content of polar groups increased after aging and large molecular weights were generated, and that the SBS modifier was degraded during aging [22,23]. Nadjet Dehouche, through four-component analysis, found that the asphaltene and gum content of SBS-modified bitumen increased after thermo-oxidative aging, the aromatic content decreased, and the saturated fraction did not change significantly, and they finally concluded that the structural changes in the composition of SBS-modified bitumen may lead to interactions between the polymer and the asphaltene chemistry [24].

Notwithstanding the fact that the F-T damage of asphalt mixtures is now sufficiently understood by relevant researchers, more systematic and comprehensive studies are being conducted on the durability of road structures under prolonged freezing and thawing conditions at low altitudes [25]. In comparison with the environmental characteristics, the frequency of freezing and thawing is low at low altitude. Nonetheless, in high-altitude areas exposed to prolonged ultraviolet radiation, asphalt pavement layer aging is serious, and there is a significant temperature discrepancy between day and night in these high-altitude regions, which can reach up to 30 °C. The pavement structure is constantly subjected to the extreme environments of thermal expansion during the day as well as freezing and dilation at night, with these two conditions non-stop switching. Simultaneously, China’s plateau area often includes SBS as the main asphalt pavement material, but the low-temperature performance and anti-aging performance of SBS-modified asphalt are slightly insufficient to meet the low-temperature crack resistance and durability requirements of asphalt pavement at high altitudes. Under the repeated action of vehicle load, it brings about cracks and aggregate spalling as well as other drawbacks of asphalt pavements, which seriously affects the service life of asphalt pavement materials in plateau areas.

On that account, a representative section of asphalt pavement in a plateau area was investigated, and the temperature within the pavement structure was monitored by means of buried sensors. On the basis of the UV and thermo-oxygen aging, high-frequency (HF) F-T tests were combined with this special environment in a plateau area. Subsequently, for the plateau region’s climatic characteristics of year-round low temperatures as well as frequent freezing and thawing, a new type of modified asphalt material with high adhesion, low-temperature cracking resistance, and UV aging resistance (RS-type modified asphalt) was developed by incorporating a special binder (R-P). Then, the indoor HF F-T cycle test design, by using cubic linear regression equations, was used to analyze the HF F-T damage to the material factors affecting the damage and damage law, reveal the F-T damage mechanism of the road engineering materials in the mountainous areas of the plateau, and make it clear that the performance of the material F-T damage contributes to the attenuation of the law. The research findings possess enormous significance for reducing asphalt pavement diseases and bettering the durability of asphalt pavement structures in plateau areas.

## 2. Materials

### 2.1. Asphalts

The asphalt used was representative of the original section of the plateau area—we used thermoplastic elastomer (SBS) as the asphalt pavement material, in accordance with standard JTG E20-2011 [26]. We employed various conventional performance indicators for test measurement, and the results are demonstrated in Table 1.

On the basis of SS-type modified asphalt, the addition of an R-P binder can optimize the cracking and anti-aging properties of RS-type modified asphalt. The primary modification mechanism of the R-P binder involves the dispersion of R-P binder and SBS-modifier particles within the void spaces of the asphalt structure, alongside additives and stabilizers that are interspersed with each other within the asphalt matrix. Their joint action reinforces the bonding force between these two phases, which thereby ameliorates the adhesion of RS-type modified asphalt. Concurrently, the composition of the R-P binder, when supplemented with a small amount of carbon black, can enhance the asphalt’s resistance to aging. The performance indicators for RS-type modified asphalt are outlined in Table 2.

### 2.2. Aggregates

RS/SMA-13 and SS/SMA-13 asphalt mixtures were made of basalt aggregate, whose properties are in accordance with the relevant technical standards. Basalt aggregate density is demonstrated in Table 3.

RS/AC-5 and RS/AC-20 asphalt mixtures were made of pebble aggregate, whose basic properties are in accordance with the relevant technical standards. Pebble aggregate density is demonstrated in Table 4.

## 3. Asphalt Mix Ratio Design

Using RS-type and SS-type modified asphalt, we prepared four types of graded asphalt mixtures. Four grade types were used in the median grades of current AC-5, AC-20 and SMA-13 asphalt mixtures for subsequent tests and gradation designs, as revealed in Table 5.

## 4. Experimental Program Design

### 4.1. Indoor Accelerated Asphalt Mixture F-T Damage Test Method

(1) Asphalt Pavement Temperature Survey

**Figure 1:**
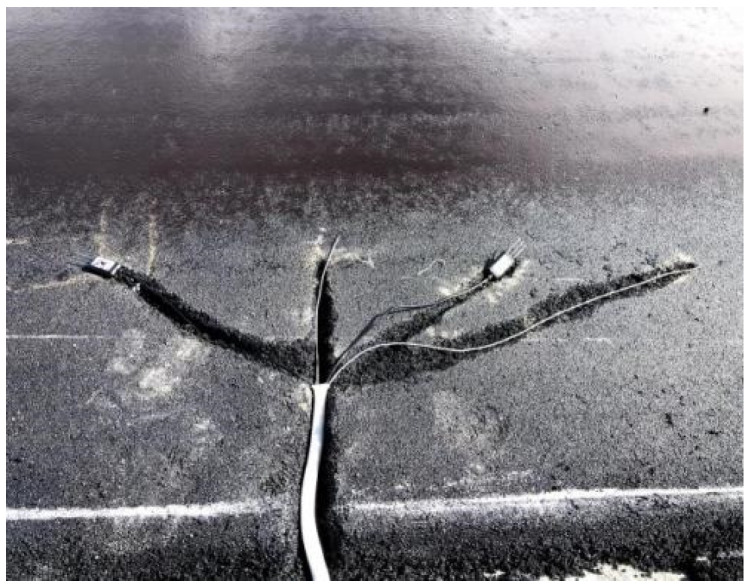
Schematic diagram of sensor deployment.

**Figure 2:**
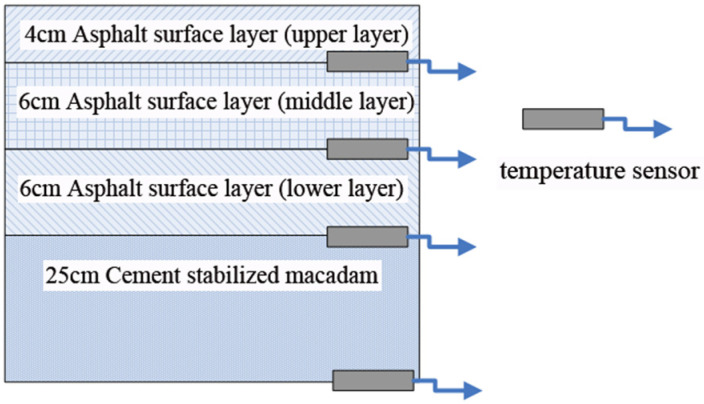
Temperature-sensor placement layers.

In order to analyze the in situ temperature of asphalt pavement in high-altitude areas, sensors for monitoring the in situ temperature of asphalt pavement were buried. These sensors are shown in Figure 1. The sensor’s buried layer is demonstrated in Figure 2.

**Figure 3:**
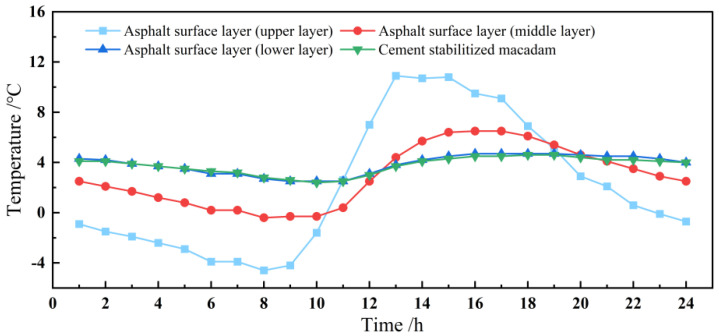
Temperatures measured in the field for each structural layer of asphalt pavement.

The asphalt pavement temperature changes and the ambient temperature were collected in real time. Taking the data of the day with the lowest temperature at the location of the road section as an example, the ambient temperature was −17 °C~2 °C, and the temperature field inside the pavement is demonstrated in Figure 3. It can be seen that the temperature field inside the asphalt pavement during the day shows a periodic sinusoidal pattern of change. The first decrease in the temperature inside the asphalt pavement in all layers is in the early morning (approximately 8:00 a.m.); this then increases from around 8:00 a.m. to 14:00 a.m., and from 14:00 a.m. to 24:00 a.m. This then reduces the law. The one-day temperature surface layer has the largest fluctuation, as the high and low extreme points are the largest; this is followed by the middle surface layer. However, the temperature field of the bottom of the layer below and the water-stabilized bottom fluctuate less and do not change much with the outside temperature. It can be seen that the temperature of the bottom of the upper layer of asphalt can be changed from negative to positive, and it will undergo the action of the F-T cycle once a day. On the bottom of the middle surface layer, the following will not occur. The depth of the pavement where the HF F-T occurs is in the range of 9.7 cm, and the bottom of the surface layer is approximately in the middle of the depth of HF F-T at the depth of 4.9 cm. Also, the low temperature of the F-T is around −3.8 °C, and the high temperature is around 10.6 °C.

(2) Indoor simulation of HF F-T test method for asphalt mixtures

Based on the actual-test temperature changes of the asphalt pavement over time, the asphalt pavement freezing temperature occurs at −3.8 °C. In the test process, to consider the cycle-shortening factors, we use the beam-temperature control temperature, and the low temperature of the core temperature is set to −5 °C. This is to achieve a short period of time between the material being in ice and then melting. So, the high temperature is controlled at 18 °C for 5~6 h for a temperature cycle, to basically realize the cycle of the asphalt mixture under the actual F-T temperature.

(3) Indoor accelerated asphalt-mixture aging test method

In addition to the HF F-T cycle, the F-T damage of asphalt pavement in plateau areas will be accelerated by the thermo-oxygen aging and ultraviolet aging of asphalt pavement in the plateau area. Therefore, the following methods are used to simulate the aging effect of asphalt pavement in a plateau environment.

Thermal-oxygen-aging test method: The prepared Marshall specimen is placed on the specimen frame and then into the 85 °C ± 5 °C blast-drying oven under forced ventilation conditions and continuous heating for 120 h ± 0.5 h. This is to simulate the asphalt pavement’s 5~7 years of service life at this degree of aging.

**Figure 4:**
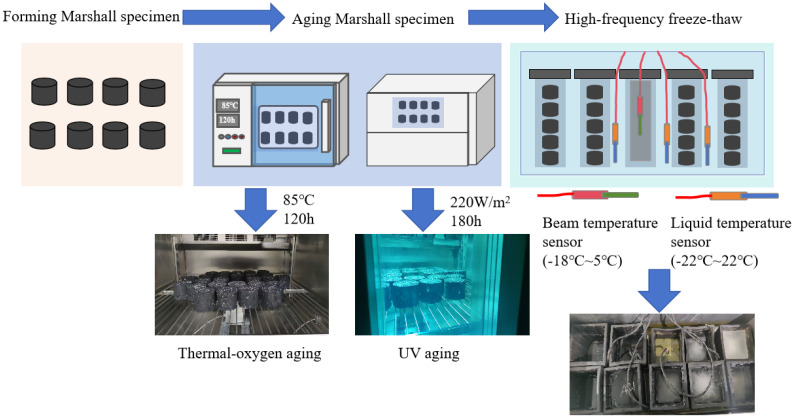
The experimental process.

UV-aging test method: We take an indoor control aging time of 180 h and an indoor UV radiation intensity of 220 W/m^2^ as the indoor UV aging conditions to simulate the natural aging of asphalt pavement outdoors over 6 months. The experimental process is revealed in Figure 4.

### 4.2. Indoor Simulation of Asphalt Mixture Resistance to HF F-T Influence Factors Test Design

The laboratory self-made highland modified asphalt is used as the research object, and the Marshall design method is used for the design of three gradation types, i.e., RS/AC-5, RS/AC-20, and RS/SMA-13 asphalt mixtures. By analyzing the increase in aging degree and the number of F-T cycles, the effect of aging conditions and the factor of maximum aggregate particle size on the resistance of asphalt mixtures to HF F-T was investigated, and at the same time, the resistance of asphalt mixture to HF F-T was compared with the performance of the top layer of SS/SMA-13 asphalt mixtures representative of the road section. The mechanical properties of asphalt mixtures under the four grades of damage, adhesion properties, and changes in volume indexes were evaluated to analyze the characteristics of F-T damage and the attenuation law of asphalt pavement materials at the macro level.

The splitting strength ratio (HTSR), mass loss ratio (ML) and void ratio variation (ΔVV) are used to describe the F-T damage suffered by asphalt mixtures under HF F-T, and their computational formulas are shown in Equations (1)–(3), respectively.

(1) $$HTSR=\frac{{\bar{R}}_{T2}}{R_{T1}}$$

where *HTSR* is the splitting strength ratio of test-group specimens; ${\bar{R}}_{T2}$ is the mean value of the splitting strength of test-group specimens after F-T; and $R_{T1}$ is the splitting the strength of specimens in the original sample group.

(2) $$ML=\frac{m_{0}-m_{1}}{m_{0}}$$

where *ML* is the rate of test-group specimens after F-T quality loss; $m_{0}$ is the dry weight of test-group specimens before F-T; and $m_{1}$ is the dry weight of test-group specimens after F-T. The dry weight of the broken asphalt-mixture specimen after HF F-T is the mass of the largest part of the specimen remaining at the time of breaking.

(3) $$\Delta VV=\left|VV_{2}-VV_{1}\right|$$

where Δ*VV* is a change in void ratio after the F-T of test-group specimens; *VV*_2_ is the void ratio after F-T of test-group specimens; and *VV*_1_ is the void ratio before F-T of test-group specimens.

In addition, it is worth noting that after the F-T cycle is completed, the Marshall specimen will remain in the void of broken ice, which will affect the results of the determination of the void ratio after F-T. In order to ensure the accuracy of the test results of HF F-T, and of the specimen void-rate test results in the determination of the value of the change in void rate, we employ the first Marshall specimen in the natural environment of natural air drying for 24 h, so that the specimen between the voids of the broken ice is fully melted. After, the specimen is placed into the Marshall specimen vacuum-drying oven so that it is dry for 48 h after the determination of its clearance rate.

The four gradation types mentioned above were used with the optimum oil-to-rock ratio, and four Marshall specimens were prepared for each gradation type (recorded as the original sample set of specimens), which were compacted 75 times on both sides so that the void-ratio range of the Marshall specimens was maintained at 4% ± 0.5%. Splitting tests were carried out according to the test protocol, and the results are summarized in Table 6.

Four different test conditions were constructed, 32 Marshall specimens (recorded as test-group specimens) were prepared with the same number of compacting times for each gradation, and the test-group specimens were subjected to F-T cycles ranging from 0 to 400 times. After 50 cycles of each freeze-thaw cycle, four Marshall specimens were selected for testing. The F-T damage of the Marshall specimens of the four gradation types after HF F-T cycles was analyzed under different conditions to study the factors influencing the F-T performance of asphalt mixtures from a macroscopic point of view. The different test conditions are demonstrated in Table 7.

## 5. Analysis of Test Results

According to the test process of the asphalt mixture, frost cracks began to appear during the F-T cycle. The entire F-T cycle process can be divided into three stages. Stage 1 is the crack formation period (less than 200 F-T cycle)—at this time, the asphalt mixture does not form more connected pores; the asphalt mixture inside the water is retained inside the material and is not easy to discharge; the formation of the freezing and expansion of the force is larger; freezing and expansion of the force occurs so that the material inside results in the production of a greater number of micro-cracks; the damage to the material is faster; and the material’s mechanical properties decay faster. In Stage 2, the crack development period (200 to 300 F-T cycles), under the action of frost expansion force, the micro-cracks further expand, forming a certain number of connected voids; there is a certain dissipation of frost expansion force; the damage caused by F-T develops slowly; and the damage rate of the material is relatively smooth. Finally, in Stage 3, the freezing-thaw damage period (300 to 400 F-T cycles), with the further F-T continuation, more connecting pores are formed and the micro-cracks inside the material further expand, causing the material damage to intensify, where the material is very likely to have larger F-T cracks or even shatter. Therefore, the third-degree polynomial fitting equation (*y* = *Ax*^3^ + *Bx*^2^ + C*x* + D) is used to analyze the F-T damage of asphalt mixtures under HF F-T cycle conditions.

### 5.1. Effects of Aging on Asphalt and Asphalt Mixtures

(1) Research on the aging mechanism of asphalt

**Figure 5:**
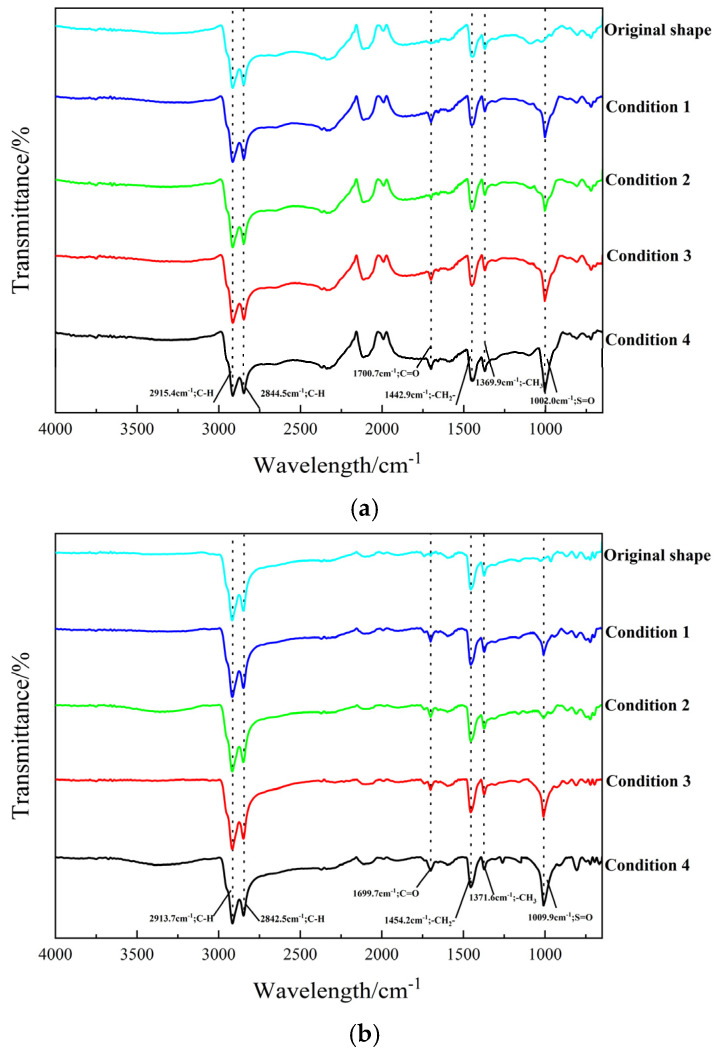
Infrared spectra of asphalt after aging. (**a**) Infrared spectroscopy of SS-type modified asphalt after aging. (**b**) Infrared spectroscopy of RS-type modified asphalt after aging.

The aging of asphalt can be grouped into thermo-oxidative aging and ultraviolet aging, which have different mechanisms but the same oxidized components detected by Fourier transform infrared spectroscopy (FTIR). As evidently suggested by associated studies, there is a remarkable correlation between the aging degree of asphalt and the peaks of carbonyl as well as sulfonyl groups in the infrared spectrum [27]. In this paper, the thermo-oxidative aging of asphalt was simulated in line with the rotating-film heating method in the standard JTG E20-2011 [26]. The UV aging of asphalt was simulated by using asphalt indoor UV illumination for 180 h. The results of the infrared spectroscopy test of the two kinds of asphalt after aging are revealed in Figure 5.

As depicted in Figure 5, the main functional groups of asphalt mainly included S=O stretching vibration (1047 to 995 cm^−1^), CH_3_ bending vibration (1390 to 1350 cm^−1^), CH_2_ bending vibration (1525 to 1395 cm^−1^), C-H symmetrical telescopic vibration (2880 to 2820 cm^−1^) and C-H asymmetric telescopic vibration (2990 to 2880 cm^−1^) [28,29]. The peaks of the carbonyl and sulfoxide groups within them both display growth after the aging of SS-type modified asphalt and RS-type modified asphalt. Nevertheless, the growth of SS-type modified asphalt was higher in contrast to that of RS-type modified asphalt. This is predominantly on account of the fact that it is easy for the SS-type modified-asphalt polar molecules to react with organic sulfides, and the chemical bond ranges from C-S-C to S=O [30]. Concurrently, in the thermo-oxygen environment, the reactive groups in the SBS polymer will be affected by the oxygen atoms, which undergo cleavage to form free radicals and finally react to form oxygen-containing chemical functional groups such as carbonyl, hydroxyl, ether, carboxyl, etc. This elevates the polarity of the asphalt component and the molecular size. The polarity and molecular size of the asphalt components are augmented. Aside from the oxidation of asphalt itself in the aging process, the R-P binder in RS-type modified asphalt is oxidized. There is evidence of desulfurization degradation and the migration of carbon black, silica and other fillers, which ameliorate the anti-aging properties of RS-type modified asphalt. Simultaneously, the augmented amount of R-P dissolved in the thermo-oxidative aging process also helps to elevate the aging resistance of RS-type modified asphalt, giving rise to RS-type modified asphalt in the carbonyl and sulfoxide; thus, the peak change is not strikingly dramatic. Nevertheless, the continuous degradation during storage will give rise to the performance deterioration of RS-type modified asphalt. Furthermore, the aging effect changes the surface structure of the asphalt and hardens its surface, which ultimately leads to the RS-type modified asphalt becoming hard and brittle.

(2) Effects of aging on asphalt mixtures

**Figure 6:**
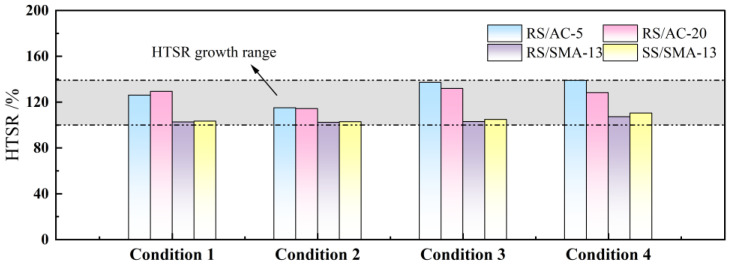
Splitting strength ratio of asphalt mixture after aging.

The above results illustrate that the aging process of modified asphalt will lessen the plasticity of modified asphalt and augment the hardness. For this reason, it will bring about an increment in the splitting strength of asphalt mixtures. In this paper, HTSR is used to describe the degree of aging of asphalt-mixture specimens subjected to aging, as illustrated in Figure 6.

As modified asphalt is in the aging process, its delay and needle penetration decrease, and the softening point augments. On top of this, the root cause is the volatilization of modified-asphalt lightweight components, resulting in a reduction of modified-asphalt plasticity and augmented hardness. As a consequence, this will cause the asphalt mixture’s splitting strength to augment. As displayed in Figure 6, all four grades of asphalt mixtures demonstrated an elevating trend in HTSR after aging. From the overall growth, the degree of its influence ranges from small to large in the following order: Condition 2 < Condition 1 < Condition 3 < Condition 4. From the standpoint of asphalt-mixture analysis, after 180 h of UV aging, the HTSR of the RS/AC-5 asphalt mixture increased to 115.1%. Subsequently, when subjected to thermal-oxygen aging for 120 h, the HTSR further elevated by 24%, marking the highest growth rate observed. In contrast, the HTSR growth of the RS/AC-20 asphalt mixture was relatively modest, reaching 128.4% under condition 4. This is predominantly attributable to the higher dosage of the RS/AC-5 in contrast to the RS/AC-20 asphalt and the smaller particle size, resulting in a thicker asphalt film on the surface of RS/AC-5 asphalt mixture. During the aging process, the asphalt film on the surface of the specimen is the most susceptible to changes. Consequently, the RS/AC-5 asphalt mixture is the most significantly affected by aging, resulting in a notable augmentation in its splitting strength. As for the RS/SMA-13 and SS/SMA-13 asphalt mixtures, the increase in HTSR was relatively small under each condition. For one thing, it is attributable to the viscosity-increasing effect of lignin fiber. In such a case, the asphalt maintains certain plastic properties after aging; in another, it can be ascribed to the fact the internal structures of the two kinds of asphalt mixtures are different. SMA asphalt mixtures employ a skeleton-dense structure, where a specific quantity of coarse aggregates forms the skeleton for this type of grading of asphalt mixtures. In accordance with the number of voids in the coarse aggregates to join the fine aggregates and fibers, it fills the skeleton voids, and the formation of a higher-density structure has high cohesion and internal friction [31,32,33]. Meanwhile, under condition 4, the HTSR of the SS/SMA-13 asphalt mixture heightened to 110.5%, while the RS/SMA asphalt mixture lessened by 2.8% in comparison, which demonstrates that RS-type modified asphalt has better anti-aging performance.

### 5.2. Effect of Maximum Aggregate Particle Size on the Resistance of Asphalt Mixtures to HF F-T Damage

**Figure 7:**
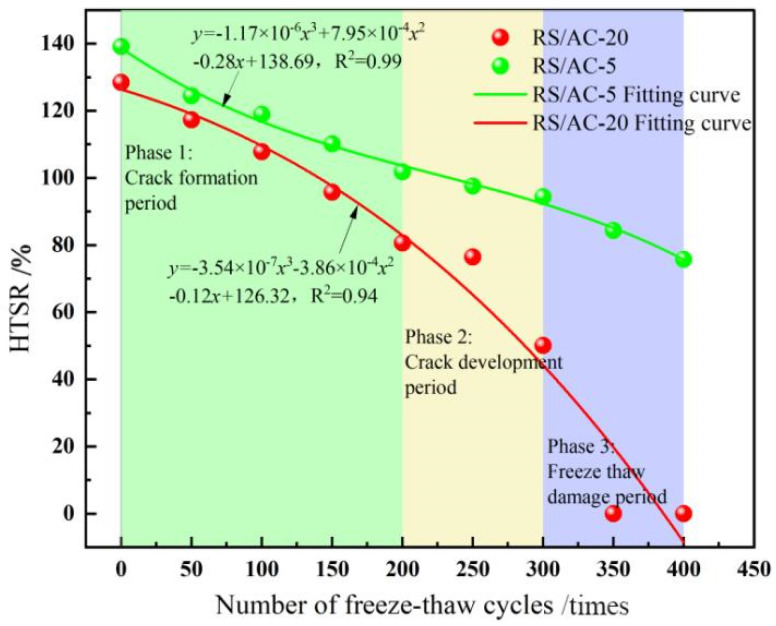
Changes of HTSR after F-T cycles of RS/AC-20 and RS/AC-5 asphalt mixtures.

**Figure 8:**
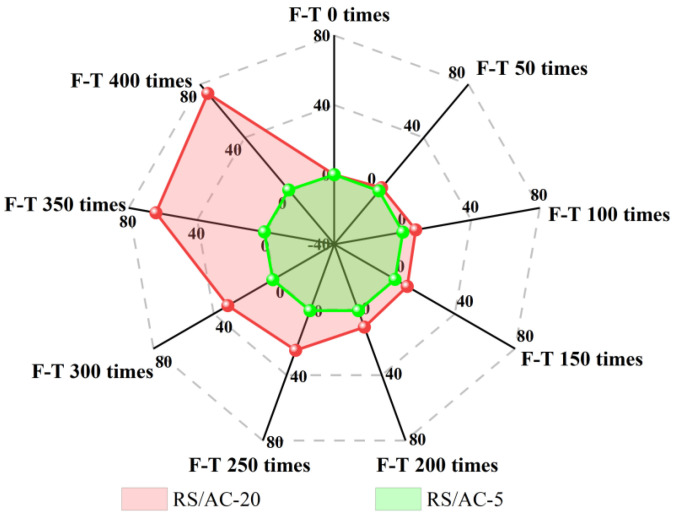
Changing law of mass-loss rate of RS/AC-20 and RS/AC-5 asphalt mixtures after F-T cycle.

**Figure 9:**
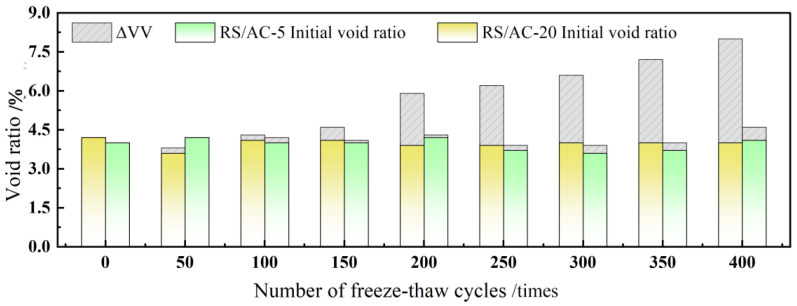
Variation rule of void ratio after F-T cycles of RS/AC-20 and RS/AC-5 asphalt mixtures.

The F-T damage of asphalt pavement is ultimately manifested as damage to the mechanical properties of asphalt mixtures. Nevertheless, associated research suggests that the maximum particle size of the aggregate is a crucial factor affecting the mechanical properties of asphalt mixtures. RS/AC-5 and RS/AC-20 asphalt mixtures are designed to probe deep into the effect of maximum aggregate size on the resistance of asphalt mixtures to HF F-T damage. Simultaneously, in line with the results obtained in the previous section, the asphalt-mixture specimens are most tremendously affected by aging under condition 4. For this reason, this paper only investigates RS/AC-5 and RS/AC-20 asphalt mixtures under condition 4, and the results are summarized in Table 8. Two kinds of asphalt mixtures after HF F-T are demonstrated in Figure 7, Figure 8 and Figure 9, respectively, referring to the HTSR, mass-loss rate and void ratio after F-T change rules.

**Figure 10:**
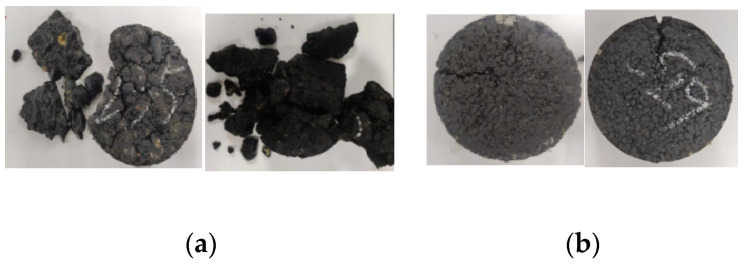
F-T damage of RS/AC-20 and RS/AC-5 asphalt mixtures. (**a**) RS/AC-20. (**b**) RS/AC-5.

As suggested in Figure 7, at stage 1, the HTSR of the RS/AC-20 asphalt mixture lowered by 22.2%, while that of the AC-5 asphalt mixture lessened by 37.4%, indicating that at the beginning of the F-T cycle, asphalt mixtures with larger aggregate sizes have more desirable mechanical properties, and their resistance to F-T damage is more exceptional. In stage 2, the gaps between the asphalt mixtures began to form connected gaps after the first 200 F-T cycles. When comparing the degree of damage to the mechanical properties of the two asphalt mixtures, the RS/AC-5 asphalt mixture exhibits relatively minor damage, and the HTSR lowered by 11.4%. However, the RS/AC-20 asphalt mixtures are relatively large, and the HTSR lessened by 56.1%. At stage 2, the larger particle sizes in the asphalt mixture make it more prone to aggregate spalling, which gives rise to the interconnected voids within the mixture transforming into larger F-T cracks [34]. This also leads to the RS/AC-20 asphalt mixture being susceptible to crushing when the F-T cycle is in stage 3, as revealed in Figure 10.

As depicted in Figure 8, a comparison is made between the RS/AC-20 and RS/AC-5 asphalt mixtures in terms of the rate at which their specimens lose mass after undergoing F-T cycles. Moreover, the mass-loss rate of the two graded asphalt mixtures also augments as the number of F-T cycles rises. In the first and second stages, the aggregate spalling of the two grades of asphalt mixtures is not tremendously conspicuous, and the maximum mass-loss rate of RS/AC-20 asphalt mixture reaches 13.5%, while the RS/AC-5 asphalt mixture reaches 0.1%, with almost no spalling of aggregates. When entering the third stage, the mass-loss rate of the RS/AC-20 asphalt mixture peaks at 73.1% under condition 4 and after 400 F-T cycles; nonetheless, the mass-loss rate of the RS/AC-5 asphalt mixture is only 5.7%. This is principally ascribable to the larger-particle-size graded asphalt mixture in contrast to the smaller-particle-size graded asphalt mixture, as its fine aggregate accounted for a smaller proportion. In environments where aging and F-T cycles frequently alternate over the long term, asphalt-mixture specimens with larger-particle-size gradations exhibit weaker bonding between asphalt and aggregates, which further heightens their susceptibility to cracking and spalling.

As suggested in Figure 9, a comparison is conducted between the extent of change in the void fraction observed in AC-5 and AC-20 asphalt-mixture specimens. After 400 F-T cycles, RS/AC-20 asphalt-mixture specimens have the largest change in void ratio, reaching 4%; the RS/AC-5 asphalt-mixture void ratio was only augmented by 0.5%. Meanwhile, in the first, second and third stages, the average change rate of the void ratio of the RS/AC-20 asphalt mixture was 0.01%/time, 0.006%/time and 0.014%/time, respectively. Nevertheless, the void-ratio change rate of the RS/AC-5 asphalt mixture remained comparatively stable. This is attributable to the larger aggregate size of the asphalt mixture, as the asphalt in the aggregate forms a relatively thin asphalt film. Aside from that, asphalt and aggregate adhesion is relatively low; hence, the specimen’s internal anti-freezing and expansion stress performance is also weaker. Under such circumstances, the growth rate of the internal void ratio in the asphalt-mixture specimens was faster, resulting in the apparent breakdown of the entire testing process for RS/AC-20 asphalt-mixture specimens. In contrast, only a small number of RS/AC-5 asphalt-mixture specimens exhibited cracks.

### 5.3. The Effect of Different Asphalt Types on the Performance of Asphalt Mixture Resistance to F-T Properties Performance

**Figure 11:**
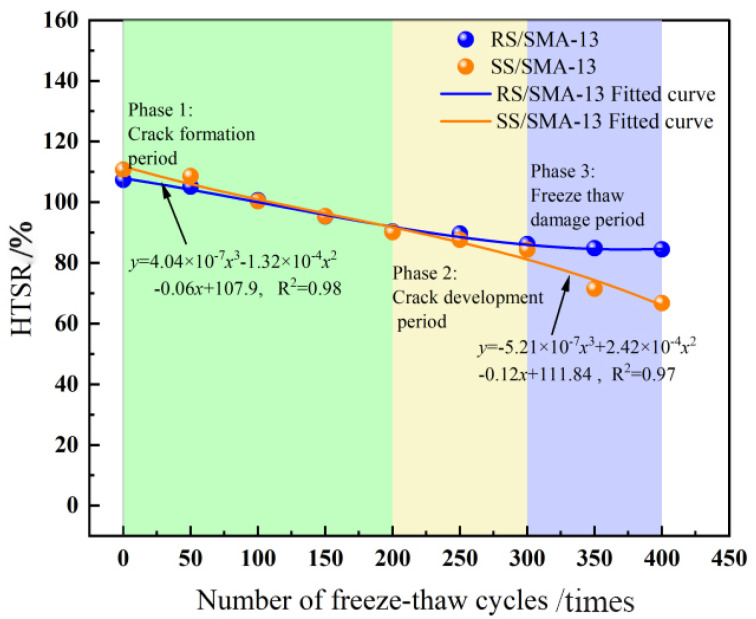
Changing law of HTSR after F-T cycles for RS/SMA-13 and SS/SMA-13 asphalt mixtures.

**Figure 12:**
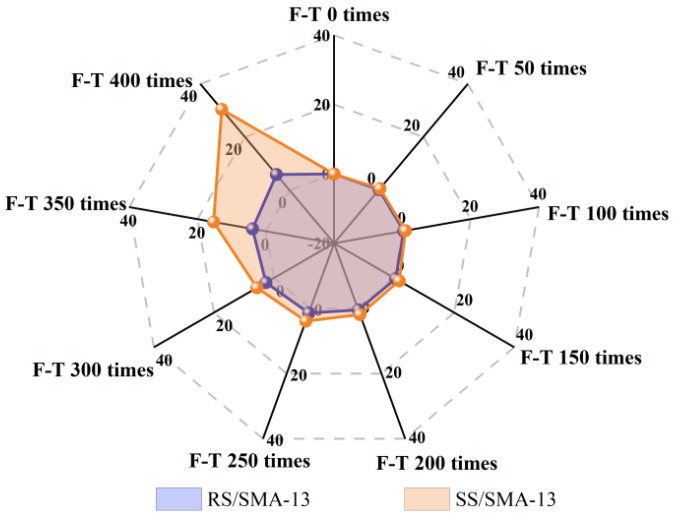
Changing law of mass-loss rate of RS/SMA-13 and SS/SMA-13 asphalt mixtures after F-T cycle.

**Figure 13:**
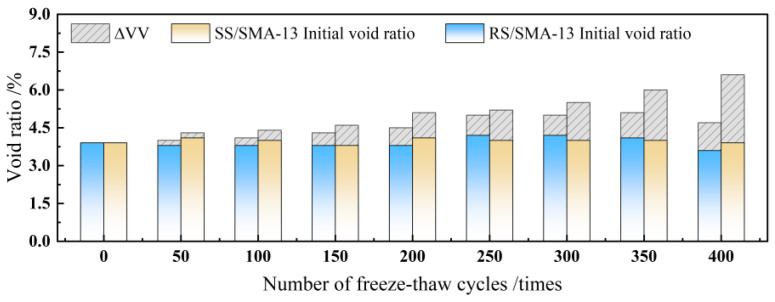
Changing law of void ratio after F-T cycles for RS/SMA-13 and SS/SMA-13 asphalt mixtures.

The SMA-13 gradation design is implemented by utilizing RS-type modified asphalt and SS-type modified asphalt, along with the original pavement material, all of which were self-made in the laboratory. The F-T resistance of the two modified asphalt mixtures was evaluated by comparing the F-T damage of RS/SMA-13 and SS/SMA-13 asphalt mixtures after F-T cycles under condition 4, and the results are summarized in Table 9. The variation patterns of HTSR, mass-loss rate and void ratio after F-T of the two kinds of asphalt mixtures after HF F-T are displayed in Figure 11, Figure 12 and Figure 13, respectively.

**Figure 14:**
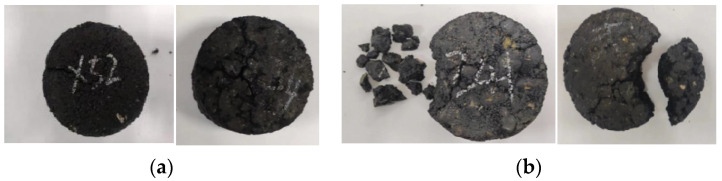
F-T damage of RS/SMA-13 and SS/SMA-13 asphalt mixtures. (**a**) RS/SMA-13. (**b**) SS/SMA-13.

As suggested in Figure 11, the HTSR of both asphalt mixtures displays a declining trend with the decrement in the number of F-T cycles. Furthermore, the HTSR of SS/SMA-13 Marshall specimens is smaller than that of RS/SMA-13 as a whole. In the first stage, owing to the light degree of F-T damages, the two kinds of asphalt mixtures are predominantly affected by UV aging and thermo-oxidizing, and the growth of both HTSRs occurs. After 200 cycles of F-T, SS/SMA-13 asphalt mixtures began to show frost cracks, and the HTSR lowered to 90.1%, at which time the distinction between the HTSR of the two asphalt mixtures was not as pronounced as anticipated. As the number of F-T cycles heightened, the cracks formed in the SS/SMA-13 asphalt mixtures expanded further at stage 2, and the HTSR was lessened by 6.8%. By stage 3, multiple cracks were formed in the SS/SMA-13 asphalt mixture, and the F-T damage was relatively severe; after 400 F-T cycles, the HTSR of the SS/SMA-13 asphalt mixture reached the lowest value, namely 66.7%; the HTSR of RS/SMA-13 asphalt mixture was relatively high, namely 84.4%; and the specimen F-T damage condition was relatively desirable as a whole. On top of that, the HTSR of RS/SMA-13 and SS/SMA-13 asphalt mixtures was relatively satisfactory. The F-T damage of RS/SMA-13 and SS/SMA-13 asphalt mixtures is illustrated in Figure 14a and Figure 14b, respectively. This is primarily a consequence of the two modified asphalts in the long aging process and low-temperature environment, as the original pavement used SS-type modified asphalt and is more prone to become brittle and have reduced viscosity reduction, resulting in unfavorable adhesion between the asphalt and aggregate, making the strength of the SS/SMA-13 asphalt mixture less desirable.

Furthermore, the mass of both asphalt-mixture specimens decreased subsequent to a long F-T cycle, which is predominantly on account of the fact that the asphalt pavement in the plateau area is in an environment of frequent F-T alternation for a long time, which not only decreases the adhesion between asphalt and aggregate but also makes the aggregate easy to peel off from the specimen. This is one of the reasons why asphalt pavements in plateau areas are prone to potholes. With the increase in the number of F-T cycles, the growth rate of the mass-loss rate of the SS/SMA-13 asphalt mixture is faster in comparison with RS/SMA-13, and the asphalt–aggregate adhesion property decreased rapidly as the F-T cycles increased [35]. As displayed in Figure 12, less-conspicuous distinction exists between the two asphalt dynamic viscosities. Aside from that, the two asphalt mixtures do not have serious F-T losses at the beginning of the F-T period; as a result, the disparity between the mass-loss rates of RS/SMA-13 and SS/SMA-13 asphalt mixtures is not immensely pronounced in the crack formation period. In the crack development stage, as the number of F-T cycles elevated, the mass-loss rate of the SS/SMA-13 asphalt mixture accelerated conspicuously. Moreover, more pronounced discrepancy existed between the mass-loss rate of the RS/SMA-13 asphalt mixture and that of the RS/SMA-13 asphalt mixture. After 200 F-T cycles, the mass-loss rate of the SS/SMA-13 asphalt mixture was 1.4% higher than that of the RS/SMA-13 asphalt mixture; the mass-loss rate of the two asphalt mixtures differed by 24.5% subsequent to 400 F-T cycles. This demonstrates that the RS-type modified asphalt can have sufficient adhesion performance with the aggregate under the environment of long aging and HF F-T.

As suggested in Figure 13, At stage 1, with the decrement in the number of F-T cycles, RS/SMA-13 and SS/SMA-13 asphalt-mixture void-ratio changes display an upward trend, but trivial discrepancy exists in the value of the change. Among them, the SS/SMA-13 void-ratio change is slightly higher than that of RS/SMA-13, which augmented by 1.0% (an average rate of change of the void ratio of about 0.005%/time). In stage 2 (F-T cycles 200 to 300 times), the asphalt-mixture void-ratio change is relatively stable compared with the RS/SAM-13 asphalt-mixture void ratio, which elevated only by 0.1%. In this stage (200 to 300 F-T cycles), the two asphalt mixtures’ void-rate change is relatively smooth, and the RS/SAM-13 asphalt-mixture void rate heightened only 0.1%. In contrast to the SS/SMA-13 asphalt mixture, the amplitude of void-rate change is 0.4% lower. This is predominantly in the F-T cycle process test with an ambient temperature below 0 °C (freezing), where the specimen void water-icing volume augmented dramatically. The internal expansion pressure within the specimen gives rise to micro-fractures. When the ambient temperature rises above 0 °C, the ice in the voids melts, triggering a reduction in volume. Relying on the inherent elastic properties of the modified asphalt, the void ratio of the specimen decreases within a certain range. For this reason, the asphalt material with ideal elastic properties will slow down the asphalt-mixture specimen void-growth rate. In the third stage, after a long time and the HF F-T cycle, the aging effect will make the modified asphalt’s original molecular chain fragmented, and there will be a disruption of the three-dimensional mesh structure formed by SBS modifiers [36]. In such a case, the modified asphalt loses some of the elastic properties; this also gives rise to SS/SMA-13 asphalt-mixture F-T damage aggravation. In the prolonged freezing and expansion of stress, the maximum void-rate change is 2.7%, and the average change rate of the void rate rises noticeably, up to 0.017%/time; in comparison with the RS/SMA-13 asphalt-mixture, the void-rate change exhibited a considerable augmentation.

## 6. Conclusions

By adopting the factors affecting the HF F-T resistance of asphalt mixtures in plateau areas, this research directs attention to how the aging environment and the maximum aggregate particle size affect an asphalt mixture’s F-T performance. Concurrently, after comparing the HF F-T resistance performance of asphalt mixtures made with laboratory-prepared RS-type modified asphalt and representative asphalt pavement-material SS-type modified asphalt, relevant conclusions were drawn below.

(1) Considering the specific environmental conditions of the pavement location, an indoor simulation method for HF F-T cycles, tailored to match in situ temperatures, was developed in accordance with existing specifications. The test conditions for this method are outlined as follows: the low temperature is −5 °C, the high temperature is 18 °C, and 5 h~6 h is a cycle. Using these aspects, the effect of HF F-T on asphalt mixtures in plateau areas is successfully simulated.

(2) Aging is a pivotal factor affecting the HF F-T damage of asphalt mixtures. The smaller the aggregate maximum particle size of the asphalt mixture, the greater the impact of aging. Among them, under the condition of 180 h of UV aging first and 120 h of thermal oxidation aging later, the HTSR of four kinds of asphalt mixtures changed at the greatest speed and amplitude. Apart from that, “180 h of UV aging, 120 h of thermal oxidation aging, 400 times of HF F-T, and splitting strength test” constitutes the most adverse simulation scenario for assessing the HF F-T resistance of asphalt pavements in high plateau regions.

(3) For asphalt mixtures with minimal aging or a lower number of F-T cycles, larger particle sizes tend to trigger more favorable mechanical properties. For this reason, at the onset of the F-T cycles, the RS/AC-20 asphalt mixture exhibits better mechanical properties in contrast to the RS/AC-5 asphalt mixture. Nevertheless, with the deepening of the aging degree and the decrement in the number of F-T cycles, the larger the particle size of the asphalt mixture, more likely the adhesive properties are to be impaired, and the specimen exhibited a faster rate of change in its void ratio. More importantly, F-T damage is serious, which makes the RS/AC-20 asphalt-mixture specimen prone to crushing.

(4) Under aging and HF F-T conditions, SS-type modified asphalt has lowered plastic properties, heightened hardness, and weakened adhesion between the asphalt and aggregate in contrast to RS-type modified asphalt. After 400 F-T cycles, the SS/SMA-13 asphalt-mixture’s HTSR is 66.7%; the RS/SMA-13 asphalt-mixture’s HTSR is 84.4%; and RS/SMA-13 asphalt-mixture specimens’ F-T damage is more desirable as a whole. This also shows that the R-P binder improves the freeze-thaw and aging resistance of its asphalt mixtures significantly. Simultaneously, RS-type modified asphalt has a certain aging resistance, but this also confirms that RS-type modified asphalt can be used in plateau areas to deal with the HF F-T of pavement adaptability.

To sum up, the type of asphalt is an important factor affecting the resistance of asphalt mixtures to HF F-T. But, for the plateau aging effect, the asphalt grading type and aggregate maximum particle size generated by the impact of asphalt pavement materials cannot be ignored. Nonetheless, the influence of the plateau aging effect, along with the asphalt grading type and maximum aggregate particle size, on asphalt pavement materials cannot be disregarded. Preparing modified asphalt with excellent low-temperature cracking, anti-aging properties and elastic properties and other types of modified asphalt, along with employing appropriate grading types and maximum aggregate particle sizes, can tremendously elevate the HF F-T resistance of asphalt mixtures. This approach is crucial for lowering pavement distress in asphalt roads in plateau areas and optimizing the durability of asphalt pavement structures in such regions.

## Figures and Tables

**Table 1:** The properties of the SS-type modified asphalt.

Project	Unit	Test Results
Softening point (global method)	°C	83.3
Needle penetration (25 °C, 100 g, 5 s)	0.1 mm	67.4
Elongation (5 °C, 5 cm/min)	cm	43.2
60 °C power viscosity	Pa·s	235,781
5 °C elastic recovery rate	%	70.7
180 °C Brinell viscosity	Pa·s	1.774
Rotating-film heating test (163 °C, 85 min)		
Needle-penetration ratio	%	86.1
Elongation (5 °C, 5 cm/min)	cm	32.9

**Table 2:** RS-type modified asphalt conventional performance indexes.

Project	Unit	Test Results
Softening point (global method)	°C	88.6
Needle penetration (25 °C, 100 g, 5 s)	0.1 mm	65.1
Elongation (5 °C, 5 cm/min)	cm	16.7
60 °C power viscosity	Pa·s	299,966
5 °C elastic recovery rate	%	74
180 °C Brinell viscosity	Pa·s	2.362
Rotating-film heating test (163 °C, 85 min)		
Needle-penetration ratio	%	88.6
Elongation (5 °C, 5 cm/min)	cm	16.2

**Table 3:** Basalt aggregate density.

Testing Indicators	Particle Size Range/mm			
	0.075~2.36	2.36~4.75	4.75~9.5	9.5~13.2
Gross volume relative density	2.691	2.823	2.861	2.875
Apparent relative density	2.691	2.940	2.947	2.922

**Table 4:** Pebble aggregate density.

Testing Indicators	Particle Size Range/mm				
	0.075~2.36	2.36~4.75	4.75~9.5	9.5~13.2	13.2~19
Gross volume relative density	2.684	2.578	2.616	2.633	2.647
Apparent relative density	2.684	2.698	2.697	2.696	2.704

**Table 5:** Asphalt mixture gradation design.

Gradation Type	Sieve Size/mm										
	19	16	13.2	9.5	4.75	2.36	1.18	0.6	0.3	0.15	0.075
RS/AC-5	100	100	100	100	99.3	50.5	34.2	25.9	18.8	12.3	9.3
RS/AC-20	99.9	89.4	72.8	58.9	38.4	29.6	20.9	15.6	11.0	7.2	5.6
RS/SMA-13	100	100	97.5	66.5	30.0	24.0	19.0	15.5	12.5	10.0	8.0
SS/SMA-13	100	100	93.6	61.3	27.7	22.4	19.9	17.0	14.6	12.7	10.2

**Table 6:** Splitting test results of the original sample-group specimens.

Gradation Type	Oil-Rock Ratio/%	Specimen Mass/g	Air Void Ratio/%	Splitting Strength/MPa
RS/AC-5	8.8	1171.5	4.1	0.92
RS/AC-20	5.3	1191.8	4.2	0.87
RS/SMA-13	6.0	1223.8	3.7	1.00
SS/SMA-13	5.7	1210.5	3.9	0.95

**Table 7:** Different test cases corresponding to the test conditions.

Test Conditions	Case 1	Case 2
Condition 1	Thermal-oxygen aging 120 h	/
Condition 2	UV aging 180 h	/
Condition 3	Thermal-oxygen aging 120 h	UV aging 180 h
Condition 4	UV aging 180 h	Thermal-oxygen aging 120 h

**Table 8:** RS/AC-5 and RS/AC-20 asphalt mixtures HF F-T test results under condition 4.

Gradation Type	Number of F-T Cycles/Times	Average Value			Standard Deviation (σ)	Coefficient of Variation (CV)
		ML/%	ΔVV/%	${\bar{R}}_{T2}$/MPa		
RS/AC-5	0	0.0	0.0	1.28	0.017	0.016
	50	0.1	0.0	1.14	0.022	0.021
	100	0.1	0.2	1.09	0.041	0.041
	150	0.4	0.1	1.01	0.032	0.034
	200	0.6	0.1	0.94	0.051	0.057
	250	0.6	0.2	0.88	0.065	0.072
	300	0.8	0.3	0.83	0.059	0.069
	350	0.8	0.3	0.79	0.028	0.033
	400	0.7	0.5	0.77	0.073	0.087
RS/AC-20	0	0.0	0.0	1.12	0.065	0.062
	50	2.4	0.2	1.01	0.036	0.035
	100	7.6	0.2	0.96	0.013	0.014
	150	8.5	0.5	0.94	0.062	0.068
	200	10.7	2.0	0.92	0.082	0.092
	250	24.9	2.3	0.66	0.088	0.102
	300	30.6	2.6	0.44	0.046	0.057
	350	64.1	3.2	0.00	/	/
	400	73.1	4.0	0.00	/	/

**Table 9:** RS/SMA-13 and SS/SMA-13 asphalt mixtures HF F-T test results under condition 4.

Gradation Type	Number of F-T Cycles/Times	Average Value			Standard Deviation (σ)	Coefficient of Variation (CV)
		ML/%	ΔVV/%	${\bar{R}}_{T2}$/MPa		
RS/SMA-13	0	0.0	0.0	1.07	0.024	0.022
	50	0.2	0.2	1.05	0.064	0.061
	100	0.4	0.3	1.01	0.032	0.032
	150	0.5	0.5	0.95	0.049	0.051
	200	0.5	0.7	0.90	0.050	0.055
	250	1.4	0.8	0.90	0.066	0.074
	300	2.7	0.8	0.86	0.014	0.016
	350	3.9	1.0	0.85	0.031	0.037
	400	5.9	1.1	0.84	0.051	0.061
SS/SMA-13	0	0.0	0.0	1.05	0.043	0.041
	50	0.6	0.2	1.03	0.023	0.022
	100	0.9	0.4	0.95	0.047	0.049
	150	1.7	0.8	0.91	0.034	0.037
	200	1.9	1.0	0.90	0.069	0.077
	250	3.9	1.2	0.86	0.031	0.036
	300	5.7	1.5	0.80	0.072	0.090
	350	15.3	2.2	0.68	0.043	0.064
	400	30.4	2.7	0.59	0.019	0.033

## Data Availability

The original contributions presented in this study are included in the article. Further inquiries can be directed to the corresponding author.
